# Two proteases with caspase‐3‐like activity, cathepsin B and proteasome, antagonistically control ER‐stress‐induced programmed cell death in Arabidopsis

**DOI:** 10.1111/nph.14676

**Published:** 2017-07-04

**Authors:** Yao‐Min Cai, Jia Yu, Yuan Ge, Aleksandr Mironov, Patrick Gallois

**Affiliations:** ^1^ School of Biological Sciences Faculty of Biology, Medicine and Health University of Manchester Michael Smith Building, Oxford Road Manchester M13 9PT UK; ^2^ College of Marine Life Science Ocean University of China No. 5 Yushan Road Qingdao 266003 China; ^3^ EM Core Facility School of Biological Sciences Faculty of Biology, Medicine and Health University of Manchester Michael Smith Building, Oxford Road Manchester M13 9PT UK

**Keywords:** endoplasmic reticulum (ER)‐stress, ER‐stress‐induced death (ERSID), plant protease, proteasome subunit PBA1, tunicamycin, unfolded protein response (UPR), vacuolar processing enzyme (VPE), vacuole

## Abstract

- Programmed cell death (PCD) induced by endoplasmic reticulum (ER) stress is implicated in various plant physiological processes, yet its mechanism is still elusive. An activation of caspase‐3‐like enzymatic activity was clearly demonstrated but the role of the two known plant proteases with caspase‐3‐like activity, cathepsin B and proteasome subunit PBA1, remains to be established.
- Both genetic downregulation and chemical inhibition were used to investigate the function of cathepsin B and PBA1 in ER‐stress‐induced PCD (ERSID). Transcript level and activity labelling of cathepsin B were used to assess activation. To study tonoplast rupture, a plant PCD feature, both confocal and electronic microscopies were used.
- Cathepsin B downregulation reduced reactive oxygen species (ROS) accumulation and ERSID without affecting the induction of the unfolded protein response (UPR), but downregulation of PBA1 increased UPR and ERSID. Tonoplast rupture was not altered in the cathepsin B mutant and cathepsin B activation was independent of vacuolar processing enzyme (VPE). VPE activity was independent of cathepsin B.
- ERSID is regulated positively by cathepsin B and negatively by PBA1, revealing a complex picture behind caspase‐3‐like activity in plants. Cathepsin B may execute its function after tonoplast rupture and works in parallel with VPE.

## Abstract 2

See also the Commentary on this article by https://doi.org/10.1111/nph.15156.

## Introduction

Plant programmed cell death (PCD) is a conserved pathway contributing to processes as diverse as development, host–pathogen defence and abiotic stress responses. One example of an abiotic stress response in plants making use of PCD is the unfolded protein response (UPR) triggered by a prolonged stress of the endoplasmic reticulum (Liu & Howell, 2010; Howell, 2013). The endoplasmic reticulum (ER) is an organelle in which protein synthesis, folding, modification and secretion take place. For a cell, it is critical to maintain protein homeostasis inside the ER. When protein homeostasis is perturbed by, for instance, a large increase in protein synthesis or a disruption of protein N‐glycosylation by the antibiotic tunicamycin (Tm), extensive protein misfolding will overload the ER capacity, resulting in ER stress (Liu & Howell, 2010; Howell, 2013). To cope with this stress, the cell will first induce several processes coming under the term UPR. The expression of several genes (such as *BIP* and *PDI*) will be induced to help restore the ER protein‐folding capacity and quality control. In addition, misfolded proteins are extracted from the ER to be degraded in the cytosol by the proteasome (ER‐associated degradation, ERAD) or are degraded by autophagy of the ER (Liu & Howell, 2016). However, if these pathways fail to reduce ER stress, a prolonged ER stress will eventually activate the PCD pathway and the affected cell will self‐destruct (Liu & Howell, 2010; Cai *et* *al*., 2014). Evidence in plants that this death is genetically controlled rather than a toxic death by ER failure are, for example, that overexpression of the PCD‐suppressor BAX inhibitor‐1 repressed ER‐induced death (Watanabe & Lam, 2008), and that ER‐stress‐induced death was absent in a quadruple mutant of the vacuolar processing enzyme (VPE) (Qiang *et* *al*., 2012). In addition, a recent report found that a NAC transcription factor in Arabidopsis, AtNAC089, was able to initiate PCD signals during ER stress induced by Tm (Yang *et* *al*., 2014).

Over recent years, good progress has been made in studying the plant signal transduction pathway which links sensing the misfolded proteins in the ER to the induction of UPR genes in plants. The architecture of the signal network is known in some detail and in particular the plant ER‐stress signal pathways share some key components with those in animals. For example, the trans‐membrane protein IRE1 senses the accumulation of misfolded proteins in the ER and catalyses the splicing of bZIP60 mRNAs in the cytosol. Spliced bZIP60 will, in turn, be translated into a functional transcription factor that triggers downstream UPR gene expression, such as *BIPs* and *PDI* that help reduce the stress for cell survival (reviewed in Howell, 2013). What remains unclear is how plant cells are able to generate a switch that re‐orientates the UPR from a cell‐survival pathway to a cell‐destruction pathway (Howell, 2013). There are components of the ER‐stress‐induced PCD (ERSID) that are known: cytochrome c release from mitochondria (Zuppini *et* *al*., 2004), reactive oxygen species (ROS) accumulation, chromatin condensation, oligonucleosomal fragmentation of nuclear DNA and a 10‐fold increase in caspase‐3‐like activity (Watanabe & Lam, 2008; Williams *et* *al*., 2014; Yang *et* *al*., 2014). In addition, both death proteases VPE and cathepsin B have been implicated (Qiang *et* *al*., 2012; Ge *et* *al*., 2016). However, no research yet has integrated these components in the pathway. What is known in animal cells is not particularly helpful because the death‐activating branch of the UPR is not conserved in plants (Cacas, 2010). There are, however, known transcription factors that connect plant PCD and ER stress. As mentioned earlier, a NAC transcription factor, AtNAC089, is thought to mediate the activation of the PCD pathway during ER stress in Arabidopsis (Yang *et* *al*., 2014). This is based on the key observation that AtNAC089 is induced by ER stress and that ectopic overexpression of AtNAC089 was able to induce PCD, whereas downregulating AtNAC089 reduced ERSID (Yang *et* *al*., 2014). In addition, AtNAC089 overexpression induces caspase‐3‐like activity (Yang *et* *al*., 2014), an activity that is a pan‐marker of plant PCD (Rotari *et* *al*., 2005). In soybean, two NAC transcription factors, GmNAC30 and GmNAC81, can form a complex to activate the expression of VPE and mediate ERSID (Mendes *et* *al*., 2013). Moreover, overexpression of GmNAC81 in soybean was able to induce caspase‐3‐like activity and triggered ERSID (Costa *et* *al*., 2008; Faria *et* *al*., 2011). Of note, throughout these examples, caspase‐3 like activity has been linked to ERSID in several experimental systems.

The plant 20S proteasome β subunit 1 (PBA1) was shown to have caspase‐3‐like activity using biotin‐DEVD‐fmk pull‐down and activity assay in downregulated lines (Hatsugai *et* *al*., 2009). This caspase‐3‐like activity of PBA1 was further confirmed by PBA1 activity‐labelling inhibition using a caspase‐3 inhibitor (Gu *et* *al*., 2010). Although PBA1 has been shown to be required for PCD induced by Pseudomonas (Hatsugai *et* *al*., 2009), its role in ERSID has not been studied yet. We showed recently that a plant cysteine protease, cathepsin B, has caspase‐3‐like activity (Ge *et* *al*., 2016). What remain unclear are the relative contribution and the genetic interactions between cathepsin B, PBA1, the vacuolar processing enzyme (VPE) and other components of ERSID. In the present study, we carried out various experiments to progress on these questions, to understand the function of cathepsin B in ERSID using Arabidopsis and to examine the interactions between cathepsin B, PBA1 and VPE.

## Materials and Methods

### Plant material and growth conditions

In order to germinate seeds on agar plates, seeds were first immersed in 70% ethanol for 10 min followed by removal of all the ethanol and dried inside a flow bench. Surface‐sterilized seeds were resuspended in 0.2% sterile agar and kept at 4°C for at least 2 d. Agar growth medium was Murashige & Skoog (MS) medium with Gamborg's Vitamin (Duchefa, Haarlem, the Netherlands) and 1.3% Phytagel. Seeds were germinated and grown in growth cabinet (Sanyo, Gunma, Japan) at 22°C under a 16 h : 8 h, light : dark period. Plants growing in compost were kept under an 8 h : 16 h, light: dark light period for growing large leaves. The triple mutant line *atcathb♯62* was obtained from Professor Paul Birch. Ipba knock‐down lines and *vpe null* lines were obtained from Professor Ikuko Hara‐Nishimura.

### Induction of ER stress using Tm

Two days before treatment, plants were transferred from short days to 16 h : 8 h, light : dark light period cabinet (Perceval, Perry, IA, USA). Tunicamycin from a Dimethyl sulfoxide (DMSO) stock was dissolved in milliQ water to a final concentration of 15 μg ml^−1^ and infiltrated with a syringe into leaves of 5‐wk‐old Arabidopsis plants grown in short days. An equivalent volume of DMSO was added to milliQ water as mock treatment. ER stress and mock treatments were infiltrated in adjacent halves on each side of the mid‐rib of the same leaf. For co‐infiltration of inhibitors with or without Tm, all chemicals were dissolved in the same solution (1 mM CA074, 50 μM β‐Lactone). For seedling treatments, seeds were germinated directly on solid MS medium supplemented with Tm at 0.2 μg ml^−1^ or mock treatment with an equivalent volume of DMSO.

### Activity labelling

Leaf tissue was ground at 4°C in extraction buffer (3 mM DTT and 100 μM PMSF). Crude extracts were centrifuged at 4°C, 18 000 ***g*** for 10 min. The supernatant was collected for labelling assays. The protein extracts were incubated with cathepsin B assay buffer (NaOAc 25 mM, NaCl 100 mM, EDTA 1 mM, pH 5.3), 3 mM DTT and 100 μM biotin‐DEVD‐FMK for 1 h at 37°C. The labelled proteins were separated by SDS‐PAGE and visualized by Western blot using a high‐sensitivity‐streptavidin‐HRP (1/20 000) (Pierce, Waltham, MA, USA).

### Enzymatic assay

For caspase‐3‐like activity assays, total proteins were extracted in extraction buffer (3 mM DTT and 100 μM PMSF) at 4°C. After centrifugation at 4°C, 18 000 ***g*** for 10 min, protein extracts were incubated in DEVDase assay buffer (NaOAc 25 mM, NaCl 100 mM, EDTA 1 mM, pH 5.5), 3 mM DTT and 50 μM ac‐DEVD_2_‐Rh110 (Bachem Ltd, Bubendorf, Switzerland). For VPE activity, the extraction and assay buffer were as for DEVDase activity except that 100 mM DTT was added in the assay buffer. The substrates were 200 μM Ac‐YVAD‐AMC (Bachem Ltd) and 200 μM Ac‐ESEN‐AMC (Peptide Institute, Osaka, Japan). For proteasome activity assays, leaves were harvested and homogenized in 20S proteasome activity assay buffer (Tris‐HCl 50 mM, MgCl_2_ 1 mM, KCl 25 mM, NaCl 10 mM, ATP 5 mM, pH 7). After centrifugation at 4°C, 18 000 ***g*** for 10 min, the supernatant was collected and 50 μM LLVY‐AMC (Enzo Life Sciences, Exeter, UK) was added to the protein extract. Cathepsin B and PBA1 activities were repressed by 1 mM CA074 and 50 μM β‐Lactone, respectively, as inhibitors. Inhibitors were incubated with protein extracts for 30 min at 30°C before adding substrates. All reactions were carried out using black 96‐well plates, at 30°C. Enzymatic activities were measured in arbitrary fluorescence unit (FLU) using an AscentTM Microplate Fluorometer with 15 real‐time fluorescence measurements every 2 min. The slope (FLU min^−1^) and the sample protein concentration determined using the mini Bradford assay (BioRAD), were used to generate the enzymatic activity in FLU/min/mg protein.

### Gene expression analysis

Total RNA was extracted using RNAzol (Sigma) according to the manufacturer's instructions. Contaminating genomic DNA was digested using DNAse RQ1 (Promega). For +RT samples, Maxima H minus reverse transcriptase (Thermo Scientific, Paisley, UK) was used for cDNA synthesis. SensiFAST SYBR (Bioline, London, UK) q‐PCR kit was used to carry out quantitative reverse transcription polymerase chain reaction (qRT‐PCR) analysis. The absolute quantification method based on a genomic DNA standard curve was used. For each sample and primer pair, a ‐RT control (without reverse transcriptase) was used to account for genomic DNA background after DNase I digestion. Each sample had three technical triplicates and each treatment had three biological triplicates. The reference gene was UBC21 (At5g25760).

### Confocal microscopy

Seedlings were mounted onto a glass slide for microscopy with the cotyledon abaxial side facing up. To stain vacuoles, 5 μM BCECF (Invitrogen, Carlsbad, CA, USA) and 5 μM FM4‐64 (Invitrogen) were used to incubate Arabidopsis seedlings at room temperature for 1 h before being subjected to microscopy. The confocal microscopy used was a Leica SP5 (inverted). To observe mRFP and FM4‐64 fluorescence, the excitation wavelength was set to 561 nm; the emission wavelength range was set to 600–640 nm. For GFP and BCECF fluorescence, the excitation wavelength was 488 nm and the emission range was 495–540.

### Transmission electron microscopy

The samples were fixed with 4% formaldehyde + 2.5% glutaraldehyde in 0.1 M Hepes buffer (pH 7.2) and postfixed with 1% osmium tetroxide + 1.5% potassium ferrocyanide in 0.1 M cacodylate buffer (pH 7.2) for 1 h, then in 1% uranyl acetate in water overnight. The samples were dehydrated in ethanol series infiltrated with TAAB Low Viscosity resin and polymerized for 24 h at 60°C. Sections were cut with a Reichert Ultracut ultramicrotome and observed with FEI Tecnai 12 Biotwin microscope at 100 kV accelerating voltage. Images were taken with an Orius SC1000 CCD camera (Gatan, Pleasanton, CA, USA).

### Ion leakage measurement

For each leaf infiltration at each time point, three leaf discs were punched out using a 4‐mm diameter cork borer in such a way as to sample the whole infiltrated area. For each leaf treatment, the three leaf discs were floated in a single well (24 well‐plate) on 800 μl deionized water for 1 h at room temperature. The conductivity of the water was then read using a B‐771 LAQUAtwin Compact Conductivity meter (Horiba, Kyoto, Japan). Data for three biological replicates were collected for each treatment.

### DAB staining

Arabidopsis leaves with appropriate infiltration treatments were detached at designated time points. Leaves were submerged in 1 mg ml^−1^ 3,3′‐diaminobenzidine (DAB) solution in a universal tube. The DAB solution was prepared according to Daudi and O'Brien (2012), at 1 mg ml^−1^ in 10 mM Na_2_HPO_4,_ pH 6.8 (no Tween‐20). Vacuum was applied for 1 min to infiltrate the leaves and then the tube was wrapped with foil and incubated for 6–8 h at room temperature. Background colour was removed by incubating leaves with 10 g ml^−1^ chloral hydrate.

### Statistics

For quantitative data, data are presented as means of replicate samples. Error bars used are equal to 95% confidence interval (CI), which is calculated as *Z* × σ/√(*n*). For null hypothesis tests, Student's *t*‐test (α = 0.05) was applied.

## Results

### Both cathepsin B and PBA1 contribute to the caspase‐3‐like activity induced during ER stress

**Figure 1:**
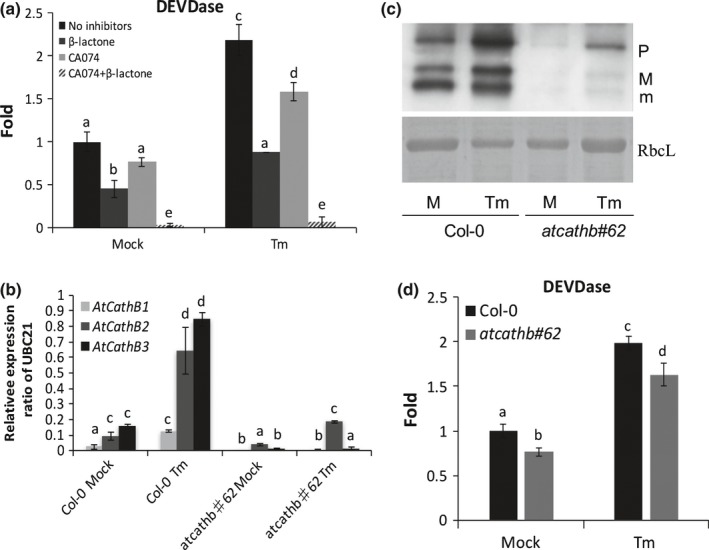
Both proteasome and cathepsin B contribute to caspase‐3‐like activity induced by endoplasmic reticulum (ER) stress in Arabidopsis leaves. (a) Caspase‐3‐like activity (DEVDase) in *Arabidopsis thaliana* leaf extract was measured using 50 μM DEVD‐Rh110 at Day 3 after mock (no inhibitor) or 15 μg ml^−1^ tunicamycin (Tm) treatment. β‐lactone (50 μM) was added to extracts to inhibit PBA1 and CA074 (1 mM) from inhibiting cathepsin B. Activity is presented as relative fold change against the Col‐0 mock sample. Error bars are 95% confidence interval (CI) of three biological replicates. Letters a–e represent groups with significant differences (*P* < 0.05; Student's *t*‐test). (b) Transcript levels of the three cathepsin B paralogues (*AtCathB1*,* AtCathB2* and *AtCathB3*). Arabidopsis Col‐0 and the triple mutant *atcathb♯62* leaves were infiltrated with mock solution or 15 μg ml^−1^ Tm. Total RNA was extracted at Day 3 post‐infiltration. Quantitative reverse transcription polymerase chain reaction (qRT‐PCR) data are presented as ratio to the reference gene *UBC21*. Primers used are in Supporting Information Table S1. Error bar represent 95% CI of three biological replicates. Letters a–d represent groups with significant differences (*P* < 0.05; Student's *t*‐test). (c) Leaves of Col‐0 or of cathepsin B triple mutant (*atcathb♯62*) were infiltrated with or without 15 μg ml^−1^ Tm. Leaves were harvested 3 d post‐infiltration. Leaf protein extracts were incubated with 100 μM biotin‐DEVD‐FMK for cathepsin B activity labelling. Biotin‐labelled proteins were separated by SDS‐PAGE and transferred to a nylon membrane following by detection using streptavidin‐HRP. As a loading control, the Rubisco large subunit (RbcL) was detected by ponceau S staining. Cathepsin B forms: procathepsin B (P), mature form1 (M), mature form2 (m). (d) Caspase‐3‐like activity (DEVDase) in leaf extract from Arabidopsis Col‐0 and *atcathb#62* was measured using 50 μM DEVD‐Rh110 at Day 3 after mock or 15 μg ml^−1^ Tm treatment. Activity is presented as relative fold change against Col‐0 mock (no inhibitor) sample. Error bars are 95% CI of three biological replicates. Letters a–d represent groups with significant differences (*P* < 0.05; Student's *t*‐test).

The increase of caspase‐3‐like activity during ER stress has been documented previously (e.g. Zuppini *et* *al*., 2004). To investigate whether both cathepsin B and PBA1 contribute the caspase‐3‐like activity in ERSID, their inhibitors, CA074 for cathepsin B (Ge *et* *al*., 2016) and clasto‐Lactacystin β‐Lactone (β‐lactone) for PBA1 (Hatsugai *et* *al*., 2009), were used to examine the contribution of these two proteases known to be responsible for caspase‐3‐like activity in plants. Tunicamycin was used to induce ER stress and caspase‐3‐like activity was measured at 3 d. Applying CA074 reduced *c*. 30% caspase‐3‐like activity in both ER stress and non‐ER stress conditions (Fig. 1a). Meanwhile, applying β‐lactone reduced caspase‐3‐like activity by *c*. 60% (Fig. 1a). These results confirmed that both cathepsin B and PBA1 were responsible for the caspase‐3‐like activity increase in our ER‐stress system. In addition, the transcript level of cathepsin B was examined. All three cathepsin B paralogues had up to a six‐fold increased transcription under Tm treatment, and these increases were abolished in the cathepsin B triple mutant line (*atcathb♯62,* McLellan *et* *al*., 2009), except for a small two‐fold increase of *AtCathB2* (Fig. 1b); *AtCathB2* is downregulated by an RNAi approach. However, this increase was much lower than that in Col‐0 Tm treated samples (Fig. 1b). Cathepsin B activity labelling using biotin‐DEVD‐fmk confirmed an increased enzymatic activity in Col‐0 3 d after treatment (Fig. 1c). In addition, the activated forms of cathepsin B were already present in the untreated sample. In *atcathb♯62,* as expected, a strong reduction of the labelling was visible in both treated and untreated samples (Fig. 1c). The DEVDase activity measured with the substrate DEVD‐Rh110 in *atcathb♯62* was only partially downregulated, in line with the cathepsinB inhibitor data (Fig. 1a). This demonstrated that cathepsin B activity was induced by ER stress at the transcript level.

### Cathepsin B is a positive regulator of ERSID in Arabidopsis seedlings

**Figure 2:**
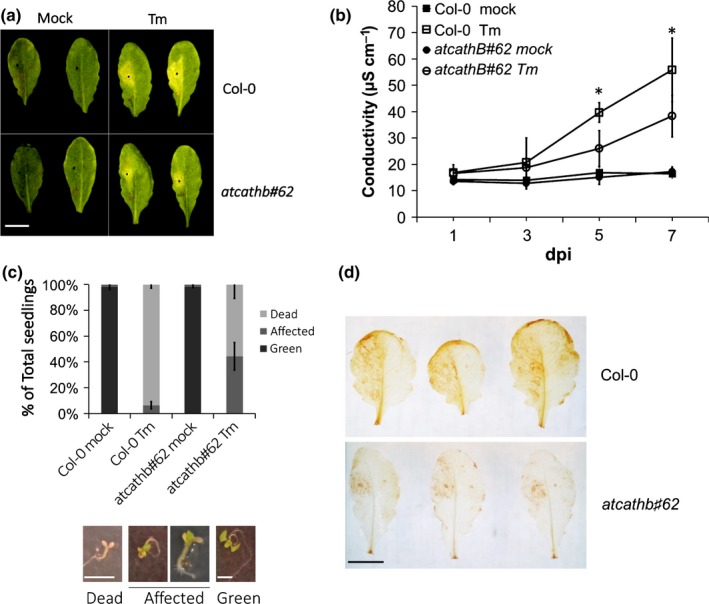
Reduced programmed cell death (PCD) phenotype of an Arabidopsis cathepsin B triple mutant under ER stress. (a) Arabidopsis Col‐0 and cathepsin B triple mutant (*atcathb♯62*) leaves were treated with 15 μg ml^−1^ tunicamycin (Tm) or mock solution on the left half of each leaf. Photos were taken 3 d after treatment. Yellow area indicates leaf cells undergoing chlorosis. Two representative leaves for each treatment are presented. Bar, 6 mm. (b) For each leaf, the mean of the ion leakage (conductivity) for three leaf discs combined was recorded at the indicated days post‐infiltration (dpi). Error bars represent 95% confidence interval (CI) of three biological triplicates. Asterisks indicate statistical significance (*P* < 0.05; Student's *t*‐test). (c) Col‐0 and *atcathb♯62* seedlings growing in Murashige & Skoog medium plate containing 0.2 μg ml^−1^ Tm or mock solution. Seedlings were scored into three classes (Dead, Affected and Green) based on their phenotype illustrated in images below. The stacked bar chart represents the % of each seedling class in total seedlings (*n* = 40) under different treatments. Error bars represent 95%CI of three replicates. (d) Col‐0 and *atcathb♯62* leaves were stained using a DAB solution to detect H_2_O_2_. The left half of each leaf was infiltrated with 15 μg ml^−1^ Tm, the other half was infiltrated with mock solution. Three representative leaves for each treatment are presented. Bar, 5 mm.

Because cathepsin B has been identified as a source of increased caspase‐3‐like activity in ERSID and the expression of the three paralogues went up, we characterized the role of cathepsin B in ERSID using the cathepsin B triple mutant *atcathb♯62*. Tunicamycin was used to induce ER stress by either infiltration into leaves or incorporation in MS medium used to grow seedlings. When 15 μg ml^−1^ of Tm was infiltrated into leaves, a chlorosis region appeared 3 d after infiltration. In *atcathb♯62* leaves, Tm infiltration resulted in an obviously smaller chlorosis region compared with the chlorosis region in Col‐0 (Fig. 2a). To quantitatively compare the ERSID between Col‐0 and *atcathb♯62*, ion leakage was measured at 1,3,5 and 7 d after infiltration of Tm. Ion leakage from mock‐treated leaves in both Col‐0 and *atcathb♯62* maintained *c*. 15 μS cm^−1^ and did not change significantly throughout the monitoring period (Fig. 2b). In Tm‐treated Col‐0 leaves, ion leakage increased twofold between 3 and 5 d after infiltration of Tm and reached 55 μS cm^−1^ by 7 d. However, ion leakage in *atcathb♯62* showed no significant increase at Day 5 and a two‐fold reduction compared to Col‐0 at Day 7 (Fig. 2b). Ion leakage was also measured in cathepsin B double mutant lines (atcathb2/3 and atcathb1/3); however, no significant change was observed between atcathb2/3, atcathb1/3 and Col‐0 (Supporting Information Fig. S1), indicating genetic redundancy. The survival of Col‐0 and *atcathb♯62* seedlings in the presence of Tm was also examined. Seeds germinated on MS medium with 0.2 μg ml^−1^ Tm were observed at 7 d. All seedlings were divided into three classes based on their phenotypes. ‘Dead’ seedlings showed bleached cotyledons and no true leaves. ‘Affected’ seedlings had green true leaves, although the cotyledons were white. ‘Green’ seedlings exhibited normal growth characters, including green cotyledons and true leaves (Fig. 2c). When treated with Tm, > 90% Col‐0 seedlings were dead and < 10% were affected. By contrast, only *c*. 55% *atcathb♯62* seedlings were dead and 45% seedlings were affected (Fig. 2c).

The production of ROS has been well‐documented during ERSID (Watanabe & Lam, 2008; Duan *et* *al*., 2010). ROS accumulation was examined in Tm‐treated leaves 3 d after Tm infiltration by DAB staining. The left half of each leaf was infiltrated with Tm or a control solution. Almost all of the area infiltrated with Tm showed DAB staining, indicating ROS accumulation in Col‐0 leaves. However, only a small area was stained by DAB in *atcathb♯62* (Fig. 2d). These suggested that cathepsin B was a positive regulator in ERSID upstream of ROS accumulation.

### Downregulation of PBA1 increases ERSID

**Figure 3:**
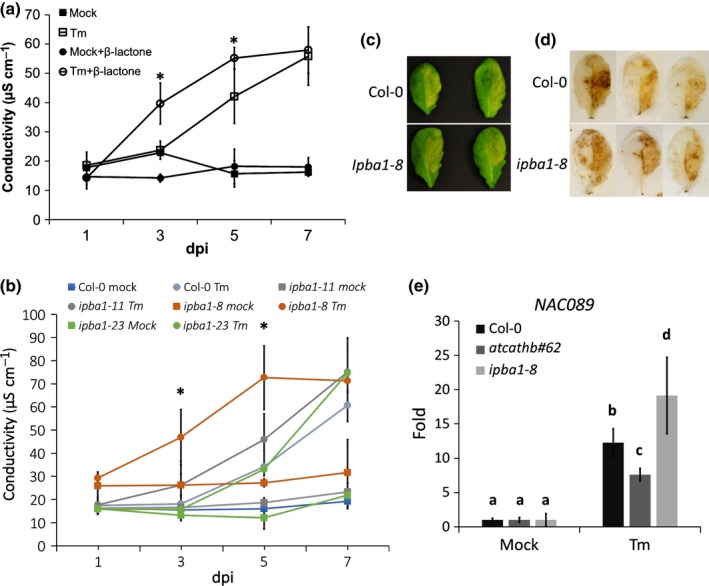
Biochemical or genetic downregulation of the proteasome increases ion leakage induced by endoplasmic reticulum (ER) stress. (a) Inhibitor. Col‐0 leaves were infiltrated with 15 μg ml^−1^ tunicamycin (Tm), 50 μM of the proteasome inhibitor β‐lactone or both. For each leaf, the mean of the ion leakage (conductivity) for three leaf discs combined was recorded at the indicated days post‐infiltration (dpi). Error bars represent 95% confidence interval (CI) of three biological triplicates. Asterisks indicate statistical significance (*P* < 0.05; Student's *t*‐test) between Tm and Tm plus β‐lactone samples. (b) RNAi lines. Leaves of Col‐0 or of three RNAi lines downregulated for PBA1 (*ipba1‐8*,* ipba1‐11* and *ipba1‐23*) were infiltrated with 15 μg ml^−1^ Tm. For each replicate, the mean of the ion leakage (conductivity) for three leaf discs combined was recorded at the indicated days post infiltration (dpi). Error bars represent 95% CI of three biological triplicates. Asterisks indicate statistical significance (*P* < 0.05; Student's *t*‐test). (c) Leaves of Arabidopsis Col‐0 and of the *ipba1‐8* RNAi line (*ipba1‐8*) were treated with 15 μg ml^−1^ Tm on the right half or mock solution on the left half of each leaf. Photos were taken 3 d after treatment. Yellow area indicates leaf cells undergoing chlorosis. Two representative leaves for each treatment are presented. (d) Col‐0 and *atcathb♯62* leaves were stained using a DAB solution to detect H_2_O_2_. The right half of each leaf was infiltrated with 15 μg ml^−1^ Tm, the left half was infiltrated with mock solution. Three representative leaves for each treatment are presented. (e) Transcript levels of the *AtNAC089* gene (*NAC089*) in Col‐0, *atcathb♯62* and *ipba1‐8* leaves infiltrated with mock solution or 15 μg ml^−1^ Tm. Total RNA was extracted at 3 dpi. Quantitative reverse transcription polymerase chain reaction (qRT‐PCR) data are presented as ratio against the reference gene *UBC21*. Primers used are in Table S1. Error bar represent 95% confidence interval (CI) of three biological replicates. Letters a–d represent groups with significant differences (*P* < 0.05; Student's *t*‐test).

The PBA1 subunit of the proteasome has been described as exhibiting caspase‐3‐like activity (Hatsugai *et* *al*., 2009) and we have shown here that it contributes to the caspase‐3‐like activity in ERSID. We therefore examined the function of the proteasome in ERSID using the cell‐permeable, irreversible, proteasome inhibitor β‐lactone at 50 μM (Hatsugai *et* *al*., 2009). Infiltrating only β‐lactone did not change the ion leakage, whereas infiltrated Tm together with β‐lactone showed a greater than two‐fold increase in ion leakage at days 3 and 5 compared to infiltrating Tm alone (Fig. 3a). It is known that β‐lactone inhibits several 20S proteasome subunits (Craiu *et* *al*., 1997). To inhibit only the PBA1 sub‐unit, three *PBA1* RNAi downregulated lines (*ipba1‐8*,* ipba1‐11* and *ipba1‐23*) were used, the same lines that were shown to have reduced proteasome and caspase‐3‐like activity (Hatsugai *et* *al*., 2009). Ion leakage of Col‐0 and ipba lines was recorded at 1, 3, 5 and 7 d post‐infiltration (dpi) of Tm. In Col‐0, ion leakage started to increase after 3 dpi to reach a two‐fold increase at 5 d. One line, *ipba1‐8*, showed clearly higher ion leakage than Col‐0, with a two‐fold increase already after 3 d, reaching a maximum at 5 d instead of 7 d with Col‐0. Line *ipba1‐11* exhibited milder but still significantly higher ion leakage than Col‐0, whereas *ipba1‐23* had a similar level of ion leakage to Col‐0 (Fig. 3b). All three *ipba1* lines and Col‐0 had similar ion leakage values at 7 d unlike the difference which was observed between Col‐0 and *atcathb♯62* (Fig. 2b). To test whether these differences correlated with different downregulation levels of *PBA1* mRNA in the lines, the *PBA1* transcript level was detected by qRT‐PCR. It showed that *ipba1‐8* had *c*. 50% of the PBA1 transcript level in Col‐0, *ipba1‐11* had 60%, whereas *ipba1‐23* still maintained *c*. 85% of the wild‐type PBA1 transcript level (Fig. S2). Therefore, the reduction in *PBA1* transcript level in *ipba* lines correlated broadly with the ion leakage data and *ipba1‐8* was chosen for further studies. ROS accumulation was examined in Tm‐treated leaves. The right half of each leaf was infiltrated with Tm and the left half with the control solution. Unlike *atcathb♯62, ipba1‐8* leaves did not show a reduced yellowing (Fig. 3c) or a reduced DAB staining (Fig. 3d). Finally, the transcript level of *AtNAC089*, a positive regulator of ERSID, was increased in *ipba1‐8* *+* Tm whereas it was induced to a lesser extent in *atcathb♯62* *+* Tm compared to Col‐0 (Fig. 3e). These data suggested that PBA1 and the proteasome are negative regulators of ERSID.

### Cathepsin B acts downstream of UPR, whereas PBA1 downregulation increases UPR

**Figure 4:**
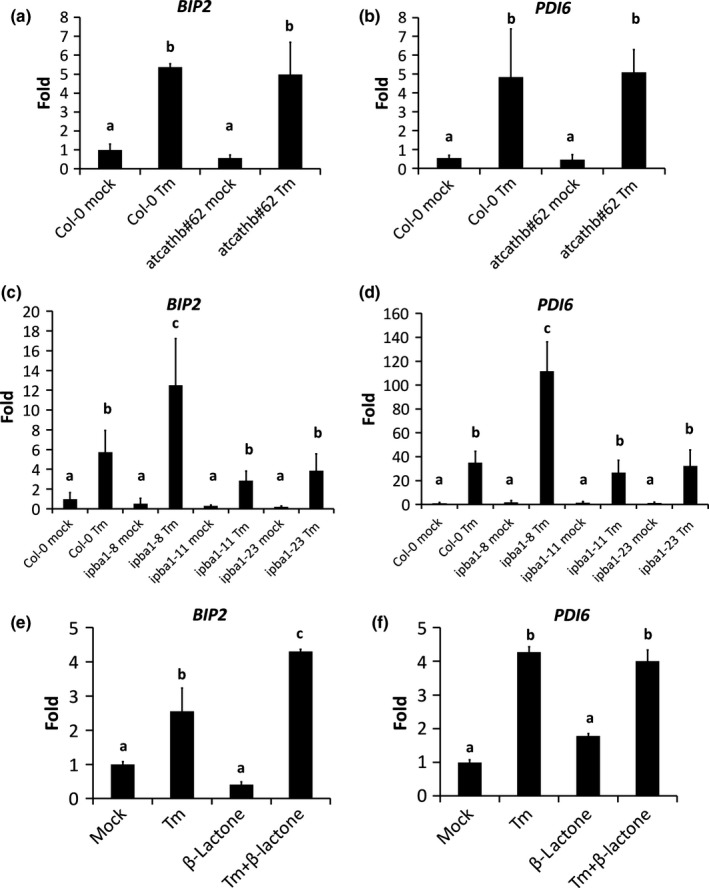
Cathepsin B and PBA1 downregulation have contrasting effects on the expression of unfolded protein response (UPR) genes. Arabidopsis leaves for each genetic background were infiltrated with mock solution or 15 μg ml^−1^ tunicamycin (Tm) and total RNA was extracted 1 or 3 d post‐infiltration (dpi). Quantitative reverse transcription polymerase chain reaction (qRT‐PCR) data for the UPR genes *BIP2* and *PDI6* are presented as a ratio of transcript level against the reference gene *UBC21*. Error bar represents 95% confidence interval (CI) of three independent leaves. Letters a–c represent groups with significant differences (*P* < 0.05; Student's *t*‐test). Primers used are in Table S1. (a, b) Col‐0 and the cathepsin B triple mutant *atcathb♯62*, 3 dpi. (c, d) Col‐0 and three RNAi lines downregulated for PBA1 (*ipba1‐8*,* ipba1‐11* and *ipba1‐23*), 3 dpi. (e, f) Col‐0 leaves treated for 1 d with mock solution and 15 μg ml^−1^ Tm with or without the proteasome inhibitor β‐lactone (50 μM).

In order to understand whether the phenotypes of *atcathb♯62* and *ipba* lines during ERSID were the result of an altered ER stress, the transcript levels of two ER stress marker genes, *PDI6* (Qiang *et* *al*., 2012) and *BIP2* (Liu *et* *al*., 2007), were compared among Col‐0, *atcathb♯62* and *ipba* lines 3 dpi of Tm 15 μg ml^−1^ and/or β‐lactone. The Tm treatment increased both *PDI6* and *BIP2* transcripts in Col‐0 and *atcathb♯62* to a similar level (Fig. 4a,b). However, *ipba1‐8* displayed a higher *PDI6* and *BIP2* transcript levels under Tm treatment, whereas *ipba1‐11* and *ipba1‐23* showed transcript levels for *PDI6* and *BIP2* similar to Col‐0 (Fig. 4c,d). When added to Tm, β‐lactone increased the transcript levels of *BIP2* at 1 dpi (Fig. 4e) but to a much lesser extent than in *ipba1‐8*, possibly because of β‐lactone degradation *in vivo*. The *PDI6* transcript level was unaffected by β‐lactone (Fig. 4f). This suggested that cathepsin B downregulation did not change ER stress/UPR, consistent with the activation of *AtNAC089* by ER stress in *atcathb♯62* (Fig. 3e). By contrast, downregulation of PBA1 in *ipba1‐8* or proteasome inhibition by β‐lactone increased ER stress/UPR**.**

### Downregulation of cathepsin B did not change proteasome activity

**Figure 5:**
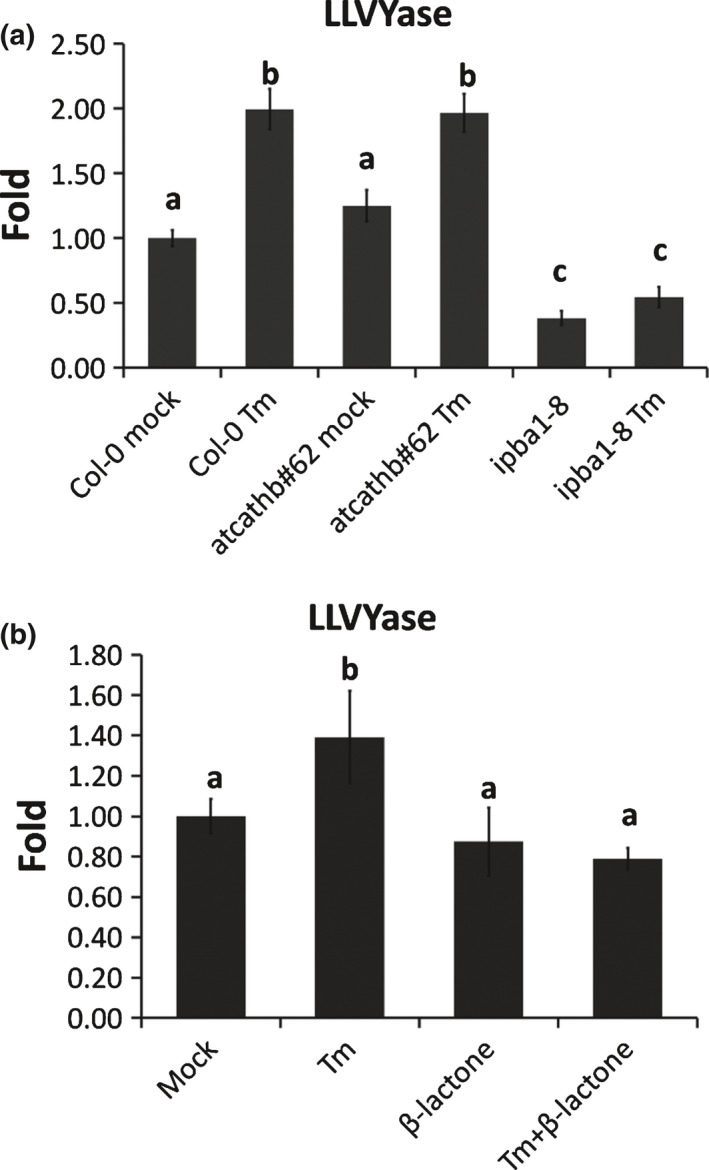
Cathepsin B downregulation does not affect proteasome activity during endoplasmic reticulum (ER) stress. Proteasome activity (LLVYase) was measured in Arabidopsis leaf protein extract using 50 μM suc‐LLVY‐AMC. Data are presented as fold change compared to Col‐0 mock. Error bars represent 95% confidence interval (CI) of three independent leaves. Letters a–c represent groups with significant differences (*P* < 0.05; Student's *t*‐test). (a) Leaves of Col‐0, the cathepsin B triple mutant *atcathb♯62 or* the *ipba1‐8* downregulated line (*ipba1‐8*) were assayed 3 d after a 15 μg ml^−1^ tunicamycin (Tm) or a mock solution (Mock) infiltration. (b) Proteasome activity (LLVYase) was measured in Arabidopsis leaf protein extract of Col‐0 using 50 μM suc‐LLVY‐AMC. Leaves of Col‐0 were assayed 1 d post‐infiltration with mock solution (Mock), 15 μg ml^−1^ Tm infiltration, mock and 50 μM of the proteasome inhibitor β‐lactone (β‐lactone) or Tm (15 μg ml^−1^) with β‐lactone (50 μM) (Tm+β‐lactone).

In order to investigate whether reduced PCD is *atcathb♯62*‐mediated via an increase in proteasome activity, we measured proteasome activity in both genetic backgrounds. The 20S proteasome activity was measured using the fluorogenic substrate Suc‐LLVY‐AMC (Hatsugai *et* *al*., 2009). There was no significant difference between Col‐0 and *atcathb♯62* as the increased activity reached the same level (Fig. 5a). As expected, LLVYase activity was reduced in the *ipba1‐8* background (Fig. 5a) and by β‐lactone infiltration in Col‐0 (Fig. 5b). This suggested that cathepsin B downregulation did not change 20S proteasome activity during ERSID.

### Downregulation of PBA1 increased cathepsin B activity

**Figure 6:**
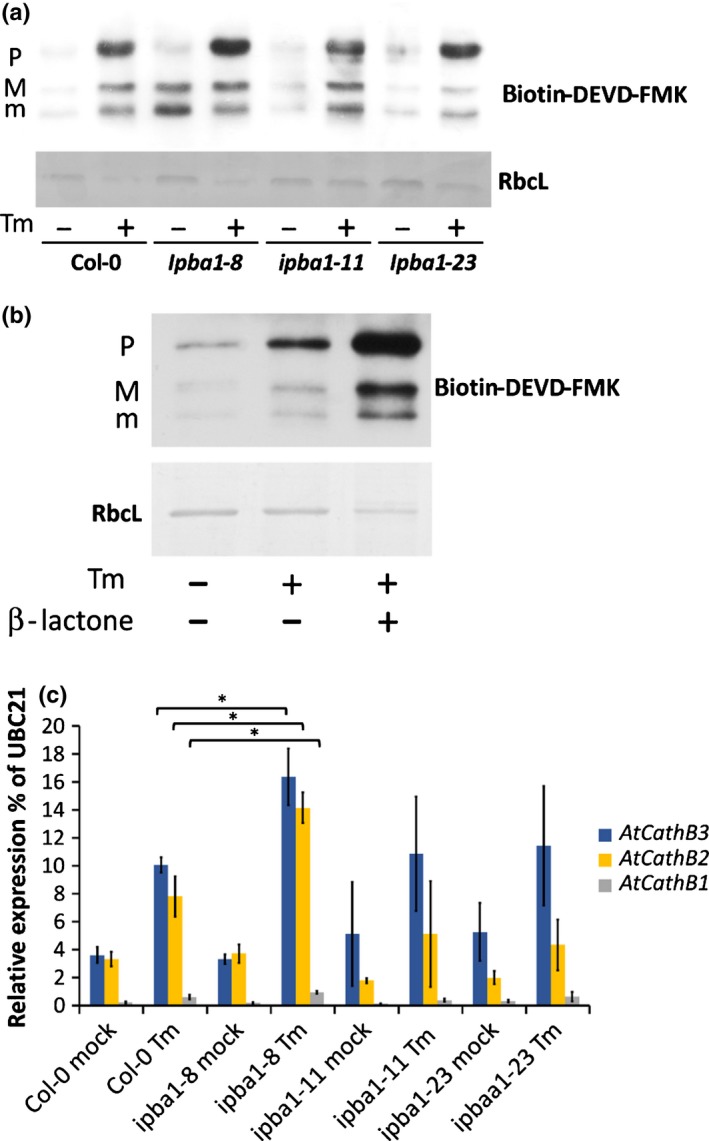
Biochemical or genetic downregulation of the proteasome does increase cathepsin B activity during endoplasmic reticulum (ER) stress. Cathepsin B forms: pro‐cathepsin B (P), mature form1 (M), mature form2 (m). (a) Cathepsin B activity labelling. Arabidopsis leaves of Col‐0 or of three RNAi lines downregulated for PBA1 (*ipba1‐8*,* ipba1‐11* and *ipba1‐23*) were infiltrated with or without 15 μg ml^−1^ tunicamycin (Tm), then harvested 3 d post‐infiltration (dpi). For cathepsin B activity labelling, leaf protein extract were incubated with 100 μM biotin‐DEVD‐FMK. After SDS‐PAGE and transfer to a nylon membrane, biotin‐labelled proteins were detected using streptavidin‐HRP. As a loading control, the Rubisco large subunit (RbcL) was detected by ponceau S staining. (b) Cathepsin B activity labelling. Leaves of Col‐0 were infiltrated mock solution, 15 μg ml^−1^ (Tm), Tm plus 50 μM of the proteasome inhibitor β‐lactone then harvested 3 dpi. For cathepsin B activity labelling, leaf protein extracts were incubated with 100 μM biotin‐DEVD‐FMK. After SDS‐PAGE and transfer to a nylon membrane, biotin‐labelled proteins were detected using streptavidin‐HRP. As a loading control, the Rubisco large subunit (RbcL) was detected by ponceau S staining. (c) Transcript levels of the three cathepsin B paralogues in Col‐0 Arabidopsis, and three RNAi lines downregulated for PBA1 (*ipba1‐8*,* ipba1‐11* and *ipba1‐23*). Leaves of each genotype were treated with or without 15 μg ml^−1^ Tm and total RNA extracted at 3 d. Quantitative reverse transcription polymerase chain reaction (qRT‐PCR) results for each gene are presented as a ratio with the control gene *UBC21* transcript level. Error bars represent 95% confidence interval (CI) for three independent leaves. Asterisks and brackets indicate the relevant statistical significance (*P* < 0.05; Student's *t*‐test).

We then tested whether repression of *PBA1*, which results in higher PCD, was able to affect cathepsin B activity. The caspase‐3 probe biotin‐DEVD‐FMK was used to label cathepsin B 3 dpi with Tm. Consistent with our previous work, three forms of cathepsin B – P, M and m, representing various stages of maturation – were labelled (Ge *et* *al*., 2016). In the absence of Tm treatment, cathepsin B activity was increased in all *ipba* lines (Fig. 6a). After Tm treatment, cathepsin B activity was increased in Col‐0 and all *ipba* lines compared with mock samples. *Ipba1‐8*, the line with the highest increase in PCD, had an increased labelling of mainly the P form of cathepsin B compared to Col‐0 (Fig. 6a), the increase of M and m was already present in untreated plants. This increase was not obvious in the remaining two lines, *ipba1‐11* and *ipba1‐23* (Fig. 6a). To confirm the increase observed in *ipba1‐8*, the activity labelling of cathepsin B was also examined in Col‐0 samples Tm‐treated in the presence of β‐lactone. Co‐infiltration of β‐lactone and Tm did phenocopy and amplify the increased labelling of cathepsin B observed when PBA1 was downregulated (Fig. 6b). To investigate whether this activity increase was due to increased transcription, the transcript levels of each cathepsin B gene were checked 3 dpi with Tm. In the mock samples, the transcript levels of the paralogues were similar across the genotypes, suggesting that the increase in cathepsin B in the absence of ER stress may be due to post‐translation regulation, especially in *ipba1‐8*. After Tm treatment, *ipba1‐8* had a 50% increase in the transcript levels of *AtCathB2* and *3*, whereas *ipba1‐11* and *ipba1‐23* showed a similar transcript level of cathepsin B compared to Col‐0 (Fig. 6c) and this correlated with the cathepsin B labelling observed in those lines. These results suggested that proteasome downregulation, whether genetic or chemical, resulted in an increase cathepsin B transcript level during ERSID that translated into higher enzymatic activity.

### Tonoplast rupture is independent of cathepsin B

**Figure 7:**
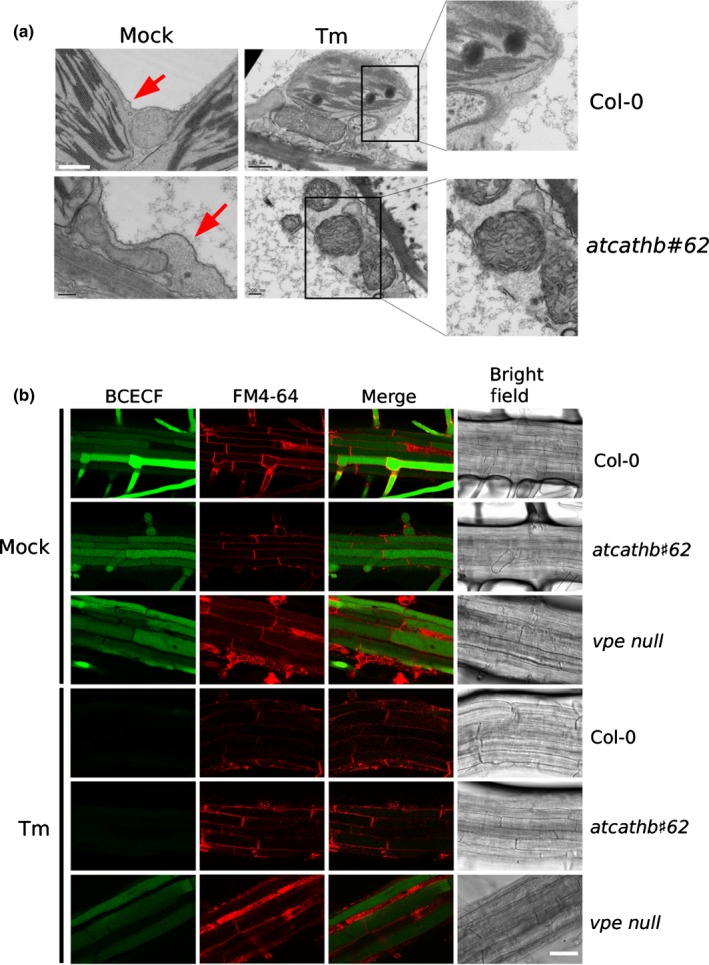
Vacuole collapse in endoplasmic reticulum (ER)‐stress‐induced programmed cell death (PCD) is independent of cathepsin B. (a) Transmission electron microscopy of representative leaf cells of Arabidopsis Col‐0 and cathepsin B triple mutant *atcathb♯62*. Leaf cells were fixed at 3 d post‐infiltration (dpi) with 15 μg ml^−1^ tunicamycin (Tm) or mock treatment. Red arrows point at the intact tonoplast. Two insets show an enlarged area of ruptured tonoplast. Bars, 200 nm. (b) Four‐day old Arabidopsis seedlings of Col‐0, *atcatgb♯62* and *vpe* null mutants were treated with or without 2.5 μg ml^−1^ Tm for 3 d. Vacuoles in root cells were stained using BCECF (green) and the membranes using FM4‐64 (red). Red, green channel, merged and white‐light confocal images of representative roots are presented. Bar, 100 μm.

Vacuole rupture has been demonstrated as an important step in many plant PCD, including during ERSID (Qiang *et* *al*., 2012). The vacuolar protease VPE has been shown to be required for vacuole rupture during virus‐induced hypersensitive response (HR) (Hatsugai *et* *al*., 2004). Cathepsin B was detected in the vacuole of Arabidopsis in vacuole proteome studies (Carter *et* *al*., 2004). In order to check whether cathepsin B was able to control the rupture of vacuole, Tm‐treated and untreated leaves from Col‐0 and *atcathb♯62* were subjected to TEM observation at 3 dpi, the time point from which ion leakage starts to rise in the wild‐type but not in the mutant. Without Tm treatment, the vacuole membrane was visualized as intact in both Col‐0 and *atcathb♯62* leaf cells (Fig. 7a). When the cells were treated with Tm, the vacuole membrane in both Col‐0 and *atcathb♯62* appeared ruptured in most cells with the organelles still intact at that stage (Fig. 7a). To rule out the possibility that the observed breaks in the tonoplast were artificially introduced during EM sample preparation, tonoplast integrity was visualized in live cells. For this, we used root cells of 4‐d‐old seedlings treated with Tm. BCECF was used to stain the vacuole, as it was demonstrated to be a good indicator for the absence of vacuolar rupture in *vpe null* studies: when the vacuole collapsed, BCECF fluorescence was switched off and this did not happen in *vpe null* protoplasts (Hatsugai *et* *al*., 2004). FM4‐64 was used to label cell membranes. In untreated root cells of Col‐0, *atcathb♯62* and *vpe null*, BCECF accumulated in the vacuole and exhibited a bright green fluorescence during the duration of the experiment (Fig. 7b). The BCECF green fluorescence in Col‐0 and *atcathb♯62* disappeared at 3 d of Tm treatment, indicative of vacuole rupture and/or a change of pH, whereas *vpe null* cells still retained the fluorescence (Fig. 7b). This indicated that unlike VPE, cathepsin B does not control vacuole rupture in ERSID.

### VPE is not required for the activation of cathepsin B and vice‐versa

**Figure 8:**
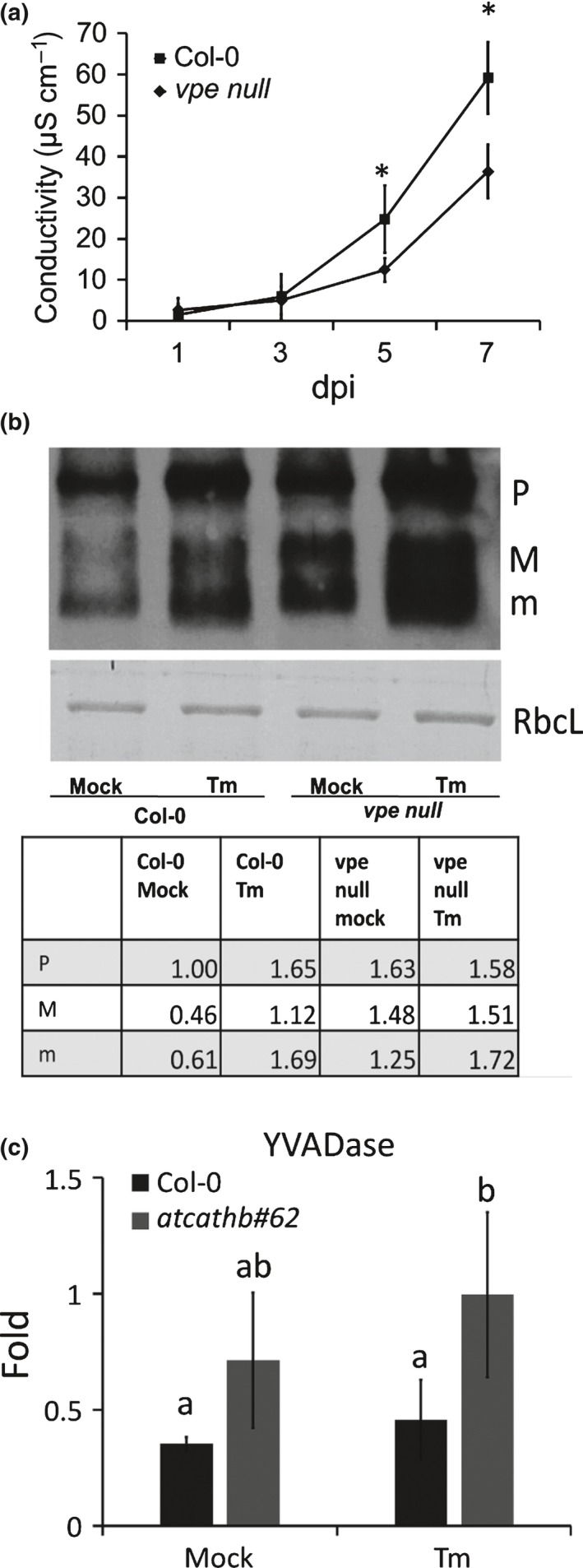
Increased cathepsin B activity labelling in *vpe null* Arabidopsis during endoplasmic reticulum (ER)‐stress‐induced programmed cell death (PCD). (a) Ion leakage of Col‐0 and *vpe* null leaves treated with or without 15 μg ml^−1^ tunicamyacin (Tm) were recorded from 1 to 7 d‐post‐infiltration (dpi) with intervals of 2 d. Tm and mock with an equivalent concentration of dimethyl sulfoxide (DMSO) were infiltrated into adjacent halves of one leaf. Three discs were punched out in each half‐leaf. The relative ion leakage was obtained by subtracting ion leakage of mock samples from Tm‐treated samples. Error bars represent 95% confidence interval (CI) of biological triplicates. Asterisks indicate statistical significance (*P* < 0.05; Student's *t*‐test). (b) Leaves of Col‐0 or of cathepsin B triple mutant (*atcatgb♯62*) were infiltrated with or without 15 μg ml^−1^ Tm, then harvested 3 dpi. Leaf protein extracts were incubated with 100 μM biotin‐DEVD‐FMK for cathepsin B activity labelling. After SDS‐PAGE and transfer to a nylon membrane, biotin‐labelled proteins were detected using streptavidin‐HRP. As a loading control, the Rubisco large subunit (RbcL) was detected by ponceau S staining. Band intensities in the table were measured using scanning and imagej (http://imagej.nih.gov/ij/) processing and expressed as a ratio of Col‐0, mock P band. All band intensities were calibrated relative to RbcL intensity as a loading reference. Cathepsin B forms: pro‐cathepsin B (P), mature form1 (M), mature form2 (m). (c) Caspase‐1‐like activity (YVADase) in leaf extract was measured using 200 μM ac‐YVAD‐AMC at day 3 after mock or 15 μg ml^−1^ Tm treatment. Activity is presented as relative fold change against Col‐0 mock (no inhibitor) sample. Error bars represent 95%CI of three biological replicates. Letters a and b represent groups with significant differences (*P* < 0.05; Student's *t*‐test).

Plant VPE is responsible for the maturation/activation of other vacuolar proteins (Hara‐Nishimura *et* *al*., 2005) and the mouse orthologous legumain has been shown to be responsible for the maturation/activation of cathepsins B, L and H in lysosomes (Shirahama‐Noda *et* *al*., 2003). We tested first whether VPEs were regulating ERSID. Ion leakage of Tm‐infiltrated leaves showed that the *vpe null* mutant had reduced PCD at days 5 and 7 (Fig. 8a), reminiscent of the *atcathb♯62* phenotype (Fig. 2b). To test whether plant VPE could be responsible for the cleavage and activation of cathepsin B in the vacuole. Cathepsin B was labelled by biotin‐DEVD‐FMK in leaf extracts, 3 dpi with Tm in leaves of Col‐0 and *vpe null*. The labelling of cathepsin B and the two mature forms of cathepsin B were present in the *vpe null* background both with and without ER stress, suggesting that VPE is not required for the maturation and activation of cathepsin B (Fig. 8b). When the relative intensities of the bands were compared using rubisco intensity as a loading control, *vpe null* leaves exhibited relative intensities similar to Col‐0 for the three forms of cathepsin B under Tm treatment, albeit an increase for the M form. However, untreated *vpe null* had more cathepsin B labelling compared to untreated Col‐0 (Fig. 8b), suggesting that the absence of VPE increases cathepsin B activity in control conditions only. Likewise, cathepsin B deficiency did not significantly affect caspase‐1 activity (YVADase, VPE) when comparing treated Col‐0 and treated *atcathb♯62* (Fig. 8c).

## Discussion

The accumulation of misfolded proteins inside the endoplasmic reticulum (ER) causes ER stress. Cells experiencing ER stress will try to restore ER homeostasis by inducing the expression of unfolded protein response (UPR) genes. If the UPR response cannot correct ER stress, a life‐death switch will eventually activate programmed cell death (PCD) (ER‐stress‐induced PCD, ERSID). Although the plant UPR gene network has been elucidated with the help of the knowledge gained from animal cells, we still have a very limited picture of the ERSID cascade in plants and particularly of the role of the caspase‐like activities involved (Cai *et* *al*., 2014). In particular, caspase‐3‐like activity, a very important PCD‐related protease activity in plant, has been found to be induced under prolonged ER stress. The correlation between plant ERSID and caspase‐3‐like activity was reinforced by the fact that the induction of this activity is under the control of AtNAC089 in Arabidopsis, a transcription factor activated by prolonged ER stress that is sufficient to trigger PCD when over‐expressed (Yang *et* *al*., 2014). In the present study, we dissected the role of two proteases responsible for caspase‐3‐like activity in the context of the PCD induced by prolonged ER stress in plants. The proteasome, and in particular its subunit PBA1, has been shown to be a source of caspase‐3‐like activity in plants (Hatsugai *et* *al*., 2009; Gu *et* *al*., 2010; Han *et* *al*., 2012) and our lab reported recently that plant cathepsin B was able to cleave the same caspase‐3 synthetic substrate (DEVD) and was strongly inhibited by caspase‐3 inhibitors and, to a lesser extent, by caspase‐1 inhibitors (Ge *et* *al*., 2016). This raised the question of how two proteases with caspase‐3‐like activity interacted during PCD. Although we showed here that both the proteasome and cathepsin B contributed to the increase in total caspase‐3‐like activity, unexpectedly the proteases had antagonistic roles: cathepsin B positively mediated PCD, whereas PBA1 negatively regulated PCD. This highlights the fact that a more complex picture than commonly presented lies beneath the correlation of increased caspase‐3‐like activity and plant PCD.

We suggest that the phenotype of PBA1 downregulation was attributed to proteasome dysfunction, as PBA1 is a critical subunit of the proteasome. Based on reports that the proteasome positively regulates PCD during HR and xylem formation (Hatsugai *et* *al*., 2009; Han *et* *al*., 2012), the downregulation of proteasome activity could have been expected to reduce ERSID. However, this was not the case in our experimental system, based on biochemical and genetic evidence. The strongest PCD increase among the three PBA1 downregulation lines used, correlated well with the strongest downregulation of PBA1. In support of this result, there is another published example of plant proteasome inhibition causing cell death. Virus‐induced gene silencing of the alpha 6 subunit of the 20S proteasome or the RPN9 subunit of the 19S, both activated PCD in *Nicotiana benthamiana*, possibly by interfering with proteasome degradation of cell‐cycle regulators in young leaves (Kim *et* *al*., 2003). The effect of proteasome inhibition on ERSID could be linked with proteasome degradation of an undetermined positive regulator of PCD as shown in other animal experimental systems (reviewed in Jesenberger & Jentsch, 2002). A preferred explanation is that the increase in PCD might be due to the increased ER stress we measured using UPR genes in the *ipba1‐8* genetic background or the proteasome chemical inhibition. Downregulation of proteasome activity is expected to reduce its ability to degrade misfolded proteins, thereby downregulating the ER‐associated protein degradation (ERAD) process. A similar increase of UPR by the application of the proteasome inhibitor MG132 has been documented in mammalian cells (Park *et* *al*., 2011).

By contrast, the downregulation of cathepsin B, reduced PCD in seedlings during prolonged ER stress, in line with what we have shown in protoplasts (Ge *et* *al*., 2016). ERSID is therefore a clear example where caspase‐3‐like activity originating from cathepsin B is required for PCD, as it is too in the PCD induced by oxidative stress (Ge *et* *al*., 2016) and during HR (Gilroy *et* *al*., 2007; McLellan *et* *al*., 2009). There is still some death occurring later in the mutant than in the wild‐type during the tunicamycin (Tm) treatment. This death could be caused by Tm toxicity to seedlings or perhaps a secondary and delayed cell death pathway that is not cathepsin B dependent. From the work on transcription factors triggering PCD due to prolonged ER stress in Arabidopsis and soybean (Costa *et* *al*., 2008; Yang *et* *al*., 2014), it can be deduced that cathepsin B acts downstream. Of note, however, is that the promoters of the cathepsin B genes, unlike those of VPE, have not been reported to interact directly with the PCD‐activating transcription factors (Costa *et* *al*., 2008; Yang *et* *al*., 2014). What strongly activates cathepsin B during prolonged ER stress remains to be discovered.

We found no evidence of a hierarchical organization between the known death proteases involved in ERSID. When proteasome activity was measured in the cathepsin B mutant, cathepsin B deficiency did not change the proteasome activity during ER stress. By contrast, repression of PBA1, chemically and genetically, increased both cathepsin B transcript and activity level. It is not simply that the proteasome affected the half‐life of cathepsin B because the transcript level of the three cathepsin B genes was increased along with the activity. The most likely explanation is that because proteasome downregulation increased ER stress and ERSID, and that ERSID induced the cathepsin B transcript level, then proteasome downregulation increased cathepsin B expression. Alternatively the proteasome could reduce the stability of transcription factors regulating cathepsin B transcription or could affect cathepsin B post‐translational regulation.

The vacuolar localization of Arabidopsis cathepsin B is reported in proteomics studies (Carter *et* *al*., 2004). The vacuolar protease VPE, that has caspase‐1‐like activity, is thought to process vacuolar proteins for maturation or activation and, in addition, caspase‐1‐like activity has been reported to be induced in ERSID (Hara‐Nishimura & Hatsugai, 2011). Because the null VPE mutant had reduced ERSID, one hypothesis was that VPE might be required for cathepsin B activation in the vacuole. However, our results showed that VPE was not required as the VPE knockout line had increased cathepsin B activity in both nontreated and Tm‐treated conditions. This demonstrated that activation of cathepsin B does not require VPE. In support of this, our previously published *in vitro* enzymatic assay showed that in an acidic pH environment such as in the lytic vacuole, cathepsin B should be able to self‐activate if the local concentration is high enough (Ge *et* *al*., 2016). Possibly, the increased cathepsin B activity may point at VPE degrading cathepsin B rather than activating it. Alternatively, a higher cathepsin B may be a compensatory mechanism for the complete loss of VPE activity in the vacuole. An additional result supporting the idea that cathepsin B is not downstream of VPE is that cathepsin B did not control the rupture of the tonoplast, unlike VPE.

Tonoplast rupture is a plant‐specific feature of vacuolar PCD (van Doorn *et* *al*., 2011) and we showed it occurring here during ERSID induced by protein misfolding, as shown also for ERSID induced by the mutualistic fungi *Piriformospora indica* in root cells (Qiang *et* *al*., 2012). Tonoplast rupture is considered a point of no return during plant PCD as degrading enzymes as expected to be released from the vacuole into the cytoplasm and degrade cellular components (Daneva *et* *al*., 2016). Interestingly, cathepsin B downregulation reduced ERSID, but did not block tonoplast rupture as VPE did. This suggests that in the cathepsin B mutant, the rupture of tonoplast did not result in PCD completion. This may not necessarily be counter‐intuitive. It is possible that cathepsin B is required to execute PCD after tonoplast rupture by activating or inactivating a key component of the pathway either in the vacuole or the cytosol. This suggestion is supported by the observation that cathepsin B was upstream of ROS accumulation during the ERSID process. Alternatively, in the absence of cathepsin B, the vacuole may not accumulate key degradative enzymes required for cell death and clearance. Or, finally, an interaction with protease inhibitors present in the cytosol may play a role, as proposed in the sentinel hypothesis for serpins controlling the consequences of the release of the protease RD21 in the cytosol (Lampl *et* *al*., 2013). This is an interesting insight in the execution stage of PCD that is coming out of our study and that deserves further investigations with various mutants.

In addition, we found that VPE activity is not affected in our cathepsin B mutant. This suggested that VPEs act in parallel to cathepsin B and the reduced cell death in the *vpe null* is probably due to a reduced vacuole rupture that prevents cathepsin B or its activated targets to reach the cytosol. The cathepsin B pathway is therefore VPE‐independent and requires vacuole rupture.

Our work contributes to the understanding of how plant proteases with caspase‐like activity fit into the plant PCD pathway and differ from the animal PCD molecular network. In particular, although animal cathepsin B may play a minor role in animal PCD (Salvesen *et* *al*., 2016), plant cathepsin B appears to be more centre stage in the process, possibly because of the prominent role of the vacuole in plant PCD. We have shown that cathepsin B and VPE seem to operate in parallel rather than in an hierarchical manner. Identifying their *in vivo* substrates will be key to understanding the events downstream of these proteases.

## Author contributions

P.G. and Y‐M.C. planned and designed the research; A.M., J.Y., Y.G. and Y‐M.C. performed experiments; and P.G. and Y‐M.C. wrote the manuscript.

## Supporting information

Please note: Wiley Blackwell are not responsible for the content or functionality of any Supporting Information supplied by the authors. Any queries (other than missing material) should be directed to the *New Phytologist* Central Office.

**Fig. S1 **Ion leakage of cathepsin B double mutant in ER‐stress‐induced PCD.
**Fig. S2** Downregulation of transcript levels for *PBA1* in *PBA1_RNAi* Arabidopsis lines.
**Table S1 **Primer sequences used in qRT‐PCR experiments
