# Supplementary material for: Two proteases with caspase‐3‐like activity, cathepsin B and proteasome, antagonistically control ER‐stress‐induced programmed cell death in Arabidopsis
Source: New Phytol. 2017 Jul 4;218(3):1143–55. doi: 10.1111/nph.14676 (PMC5947621; doi:10.1111/nph.14676)
Supplement: Supplementary file 1 — Fig. S1 Ion leakage of cathepsin B double mutant in ER‐stress‐induced PCD. Fig. S2 Downregulation of transcript levels for PBA1 in PBA1_RNAi Arabidopsis lines. Table S1 Primer sequences used in qRT‐PCR experiments [file NPH-218-1143-s001.pdf]

New Phytologist **Supporting Information**

Article title: Two proteases with caspase 3-like activity, cathepsin B and proteasome, antagonistically control ER-stress-induced programmed cell death in *Arabidopsis*

Authors: Yao-Min CAI, Jia YU, Yuan GE, Aleksandr MIRONOV and Patrick GALLOIS

Article acceptance date: 24 May 2017

The following Supporting Information is available for this article:

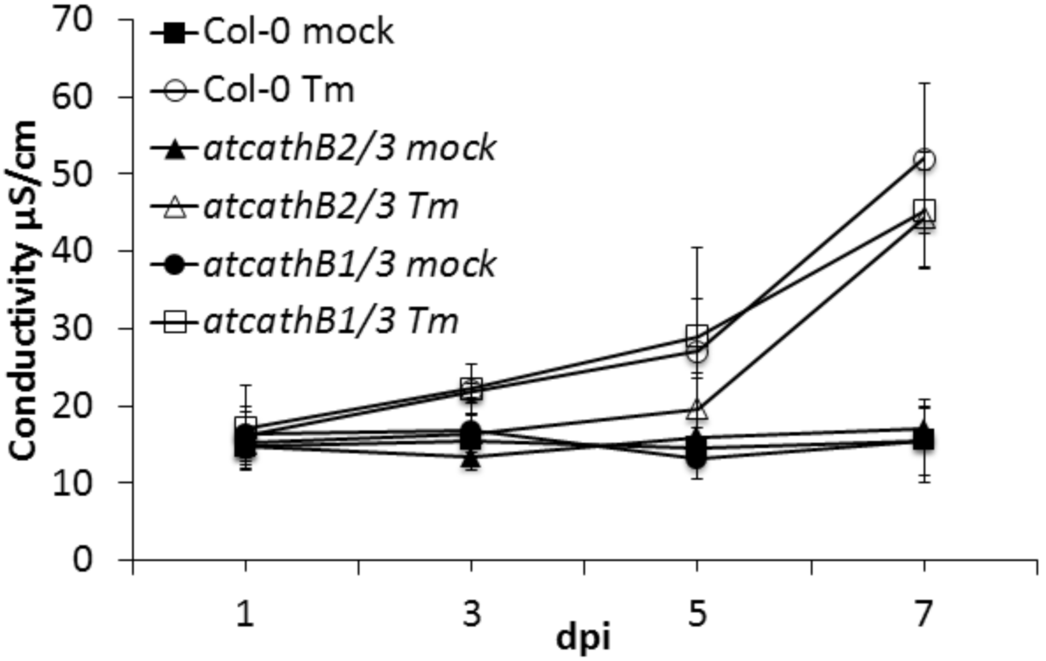

Fig. S1 Ion leakages of cathepsin B double mutants in ER stress-induced PCD. Col-0 and cathepsin B double mutants (*atcathb2/3* and *atcathb1/3*) leaves were infiltrated with 15µg/mL tunicamycin (Tm) or a mock solution (M). For each replicate, the mean conductivity of three leaf discs was measured. Data was measured at one, three, five and seven days after infiltration. Error bar=95%CI for biological triplicates.

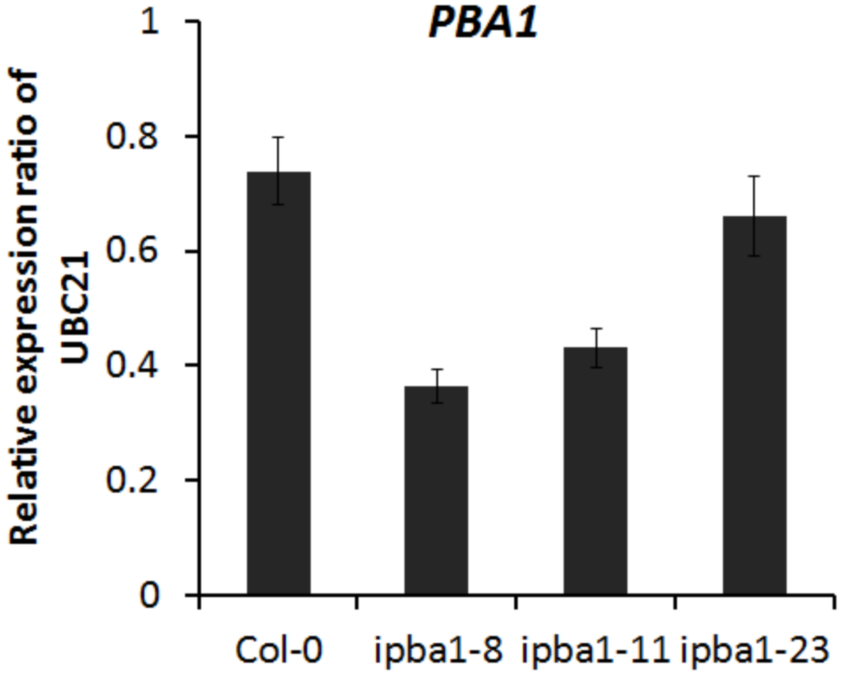

Fig. S2 Down regulation of transcript levels for *PBA1* in *PBA1_RNAi Arabidopsis* lines. Total RNA was extracted from 5-weeks-old leaves. The transcript level of *PBA1* in Col-0 and *PBA1* down-regulation lines (*ipba1-8*, *ipba1-11* and *ipba1-23*) leaves was measured by qRT-PCR using primers qPBA1-F and qPBA1-R (Table S1). Expression is represented relative to the transcript level of *UBC21*used as a reference gene. Error bar= 95%CI of biological triplicates. Hatsugai *et al*. (2009, fig 3G) has shown that these lines have the activity for their proteasome subunits PBA1, PBB and PBE reduced by 60 to 80%.

Table S1. Primer sequences and corresponding genes used for qRt-PCR experiments.

Primer ID	Gene	AGI code	Primer sequence
pNAC089_326F	AtNAC089	AT5G22290	5’-TTTCGAGCCTTGGGATTTAC
pNAC089_455R	AtNAC089	AT5G22290	5’-GCTCTTTCCCAGTTGCTTTC
SYBR-Bip2-F	Bip2	AT5G42020	5'-TCAGCACCAAGTCCGTGTAG
SYBR-Bip2-R	Bip2	AT5G42020	5'-CTTCACAGGTCCCATGGTCT
SYBR-2300-F	Cathepsin B1	AT1G02300	5'-CCACGGTGTAGTAACCCAAGA
SYBR-2300-R	Cathepsin B1	AT1G02300	5'-GTATGCGCCGACACCATAG
SYBR-2305-F	Cathepsin B2	AT1G02305	5'-GTGTTAGCGGAAACCAGCTT
SYBR-2305-R	Cathepsin B2	AT1G02305	5'-CAGTGAAGGCAACCTCAACA
SYBR-1610-F	Cathepsin B3	AT4G01610	5'-GAAATGCGTTAGCGACAACA
SYBR-1610-R	Cathepsin B3	AT4G01610	5'-CTGCCATGATATCTTGTGGATT
qPBA1-F	PBA1	AT4G31300	5'- CATGCTCCAAACTGGTCTCA
qPBA1-R	PBA1	AT4G31300	5'- CAAACGGTTGCTCGACTACA
qPDI-F	PDI6	AT1G77510	5'- TGAGAAATGGAGGGAAGTCG
qPDI-R	PDI6	AT1G77510	5'- CAACAACCTCAGTGGCAGAA
qUBC21-409F	UBC21/PEX4	AT5G25760	5’-ACAGCGAGAGAAAGTAGCAGA
qUBC21-489R	UBC21/PEX4	AT5G25760	5’-TTGATAAGAGCGGTCCATTTGAA

Reference:

Hatsugai N, Iwasaki S, Tamura K, Kondo M, Fuji K, Ogasawara K, Nishimura M, Hara-Nishimura I. **2009**. A novel membrane fusion-mediated plant immunity against bacterial pathogens. *Genes and Development* **23**: 2496–2506.
